# Acute Mental Health Impacts and Mid-Term Symptom Trajectories Following Wildfire Exposure

**DOI:** 10.3390/ijerph23081005

**Published:** 2026-07-31

**Authors:** Lusine Gigoyan, Michelle L. Dossett, Kathryn C. Conlon, Shuai Chen, Sean M. Raffuse, Irva Hertz-Picciotto

**Affiliations:** 1 Department of Public Health Sciences, School of Medicine, University of California, Davis, CA 95616, USAihp@health.ucdavis.edu (I.H.-P.); 2 Division of General Medicine and Bioethics, School of Medicine and Center for Healthcare Policy and Research, University of California, Davis, CA 95817, USA; 3 Air Quality Research Center, University of California, Davis, CA 95618, USA

**Keywords:** wildfires, mental health, natural disasters, acute and mid-term trajectory, psychological distress, observational study

## Abstract

**Highlights:**

**Public health relevance—How does this work relate to a public health issue?**

- Wildfires can cause substantial psychological distress.
- Using a symptom-based approach to understand the immediate and medium-term mental health impacts of wildfires may inform timely interventions and disaster response strategies.

**Public health significance—Why is this work of significance to public health?**

- Wildfires are increasing in size and expanding into additional geographic regions, with the smoke extending far beyond the fires themselves. As the numbers of people affected globally is rising rapidly, research to understand the health effects, both physical and mental, during and after the fires is more urgent than ever, and of mounting significance.

**Public health implications—What are the key implications or messages for practitioners, policy makers and/or researchers in public health?**

- This study highlights the need for early public health services and interventions to mitigate progression to more severe mental health conditions.

**Abstract:**

This longitudinal cohort study examined acute and mid-term mental health impacts among 4346 survivors of the 2018 Camp Fire in Northern California using a retrospective survey that asked participants to report symptoms at one, three, and six months post-wildfire. Forty-one percent of participants reported at least one mental health symptom three weeks after the wildfire. Mental health symptoms declined over the six-month follow-up. The mean number of mental health symptoms was 3.7 (95% CI, 3.3–4.1) at three weeks, 3.3 (3.0–3.7) at one month, 3.1 (2.8–3.4) at three months, and 2.7 (2.5–3.1) at six months. Among survivors reporting one or more symptoms, severe home damage was associated with a 1.6-fold higher number of mental health symptoms, preexisting mental health conditions and job loss due to the wildfire were each associated with 1.2-fold increase, and prior physical disabilities with a 1.1-fold increase. Loss of a family member or friend and living alone were also associated with a higher number of mental health symptoms, whereas both younger age (<18 years) and male sex were associated with fewer symptoms. Wildfire exposure is associated with increased mental health risks, particularly among those with preexisting conditions and significant fire-related losses.

## 1. Introduction

Climate change has substantially increased the likelihood of extreme wildfires over the past decades [1]. Between 2000 and 2024, California experienced approximately 8243 wildfires, with an average of 974,894 acres burned annually [2,3]. Climate models indicate that ongoing climate change, characterized by rising temperatures and decreasing precipitation, is likely to increase the number of days with extreme fire weather [4]. This trend is expected to lead to a higher frequency and greater intensity of wildfires in the future [4]. The escalating frequency of natural disasters has prompted an interest in understanding their impact on mental health.

A growing body of literature has examined the aftermath of traumatic events, revealing significant negative impacts on survivors’ mental well-being. Evacuation and displacement resulting from disasters often exacerbate mental health issues, particularly among vulnerable populations, manifesting in conditions such as post-traumatic stress disorder (PTSD), anxiety, and general mental distress [5,6,7,8]. Natural disasters, including wildfires, have been associated with an increased prevalence of a wide spectrum of mental health disorders, including generalized anxiety disorder (GAD), PTSD, depression, paranoia, and increased alcohol/drug use [5,6,9,10,11].

Previous wildfire-related mental health research has largely focused on specific psychiatric disorders, particularly PTSD, GAD, and major depressive disorder (MDD) [6,12,13]. Evidence from studies conducted following the 2016 Fort McMurray wildfire in Alberta, Canada, provides insight into the psychological consequences of wildfire exposure among the affected populations [14]. Compared with the general Canadian population, survivors of the Fort McMurray wildfire experienced a substantially higher prevalence of anxiety- and trauma-related symptoms [14]. Ayepong et al. reported that 15.7% of survivors met criteria for GAD, compared with an estimated prevalence of 2.4% to 3.0% in the general Canadian population [15]. The prevalence of PTSD was also elevated among survivors (10.2% vs. approximately 8% nationally) [15]. These outcomes were associated with disaster-related exposures, including witnessing homes engulfed in flames, experiencing fear during evacuation, and perceived lack of social support [14]. Evidence further suggests that these mental health effects may persist beyond the immediate aftermath of the disaster, with elevated prevalence of GAD and PTSD reported at six and 18 months post-wildfire [13]. Additional survey-based research documented high rates of insomnia (28.5%), PTSD (15%), and MDD (15%) among affected individuals [16].

Although existing studies provide important insights into the mental health burden associated with wildfire exposure, they may underestimate the true population-level impact. Individuals who do not meet formal diagnostic criteria or who do not seek clinical care are likely underrepresented in prior research, particularly in disaster settings where access to healthcare may be disrupted due to infrastructure damage, displacement, or overwhelmed health systems. Consequently, the mental health impacts of wildfire exposure—especially those occurring in persons without formal diagnosis or with subclinical symptoms—remain incompletely characterized and therefore ignored in health system-based studies.

To date, little research has focused on mental health symptoms and subclinical conditions, rather than formally diagnosed disorders, in the context of wildfires. Evidence from studies of other natural disasters suggests that subthreshold mental health symptoms are often more prevalent than diagnosable conditions and may serve as important risk factors for the subsequent development of full psychiatric disorders over time [17,18,19,20]. These symptoms can nonetheless cause substantial distress, impair daily functioning, and reduce quality of life, underscoring their public health significance [21].

Understanding the occurrence and progression of mental health symptoms in the immediate and medium-term aftermath of wildfires is therefore critical for informing early, targeted public health interventions that may mitigate progression to more severe mental health conditions. To address these critical gaps, this study aims to (1) identify risk factors associated with the development of mental health symptoms in the immediate aftermath of the wildfire; and (2) characterize the prevalence and trajectories of a broad range of mental health symptoms following the 2018 California Camp Fire. We examined across the immediate three weeks, when the wildfire was smoldering, at one month, when it was fully contained, and medium-term (three and six months) post-disaster periods, providing a more comprehensive assessment of wildfire-related mental health impacts and the trend over a six-month period.

## 2. Methods

### 2.1. Study Setting

In 2018, a series of large wildfires erupted in Northern California. These wildfires were unusual in their scale, the rapidity of their spread, and the vast areas that were affected. The Camp Fire, which started on 8 November 2018, in the town of Paradise, at the time was the most deadly and destructive in the history of California. Around 52,000 people were evacuated, approximately 9000 homes and 18,000 structures were destroyed, 150,000 acres were burned, and 85 people died [22]. In response to the 2017 and 2018 fires in Northern California, the UC Davis Environmental Health Sciences Center (EHSC) initiated a research program to better understand the health impacts of these conflagrations: ***W***ildfires and ***H***ealth: ***A***ssessing the ***T***oll in ***No***rthern ***Ca***lifornia (WHAT-Now? CA Study). This current investigation of mental health impacts is based on data collected in the WHAT-Now? CA Study.

### 2.2. Study Design and Data Collection

We conducted a retrospective cohort study of individuals who completed the online survey developed by the UC Davis EHSC. The survey was initially deployed in response to the 2017 Northern California wildfires [7] and modified for future use when the 2018 fires erupted. The analysis presented here leverages the modified version of that survey.

The survey was launched in April 2019 and was available for completion until February 2020. Most responses (85%) were completed between April and July 2019 (five to eight months post-wildfire). It included questions that retrospectively assessed survivors’ experiences and exposures related to the Camp Fire and other 2018 Northern California wildfires. The survey was publicized through social media, specifically Facebook wildfire groups, to enhance enrollment. This was a common way for individuals who were impacted by the wildfire to communicate and learn information about available resources. After completing questions regarding eligibility and indicating consent, survivors were prompted to complete the online survey.

The survey was designed to be completed by one adult per household. The respondent provided information for the entire household regarding demographics, general health status, pre-existing health conditions, evacuation details, losses, injuries, mental health status, and the onset of new symptoms following the wildfires.

### 2.3. Study Population

Inclusion criteria for the respondents were: age 18 years or older, provision of informed consent, and residence in a Northern California county at the time of the 2018 Camp Fire.

**Figure 1:**
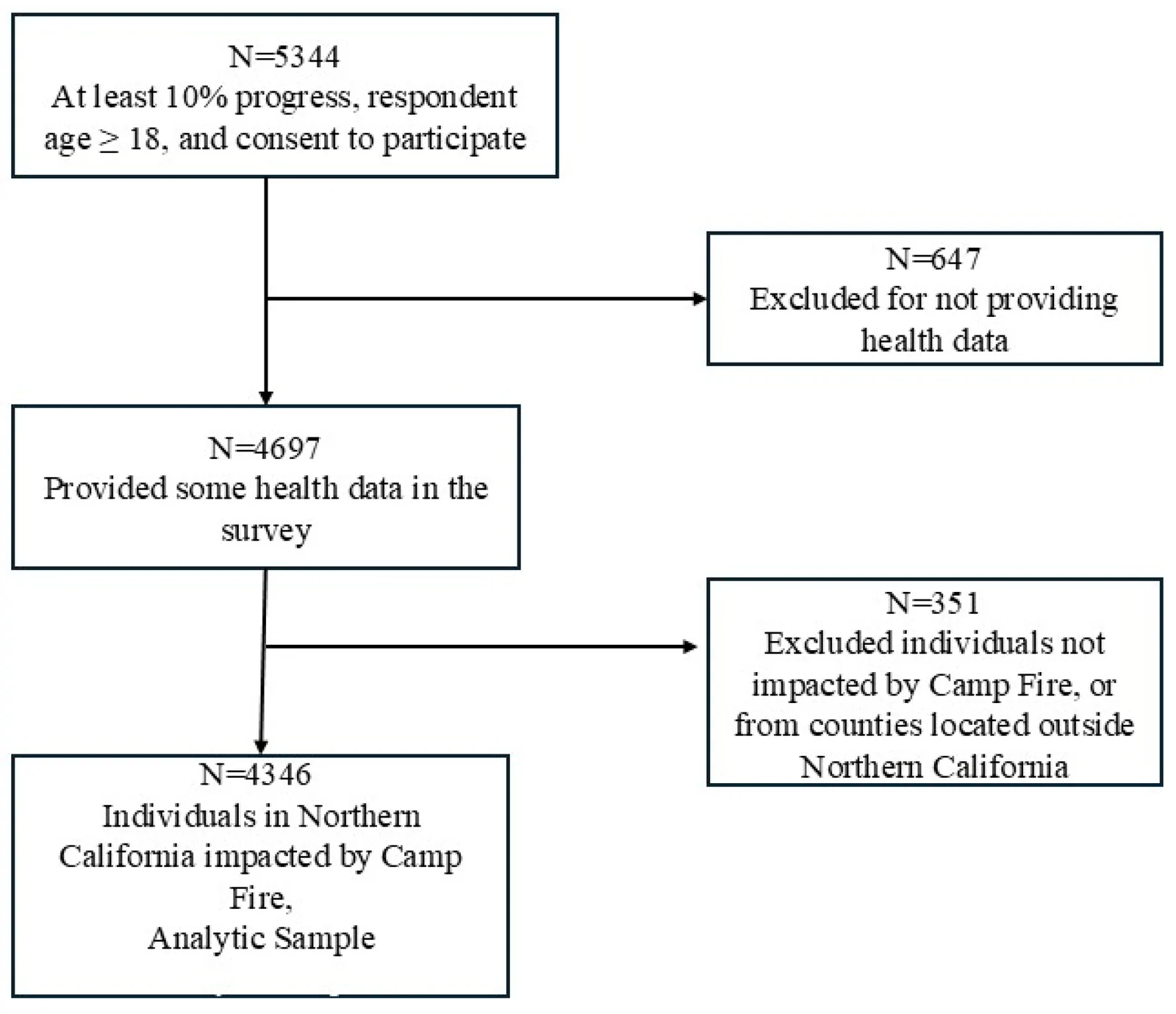
Flow Chart of Study Participants.

A total of 1639 households, representing 4346 individuals, were included in this study. A detailed flow diagram with inclusion criteria is shown in Figure 1.

The cohort included survivors from multiple affected counties in California that we defined as: Butte, Shasta, Sacramento, Siskiyou, Yolo, Sonoma, Placer, Lake, Sutter, Yuba, Mendocino, Contra Costa, Tehama, Solano, Alameda, Napa, Nevada, Marin, Trinity, El Dorado, Glenn, San Francisco, Amador, Humboldt, San Joaquin, Sierra, Plumas, and Colusa.

We further defined the study population based on elevated PM_2.5_ associated with their address for any day 8–25 November. Elevated PM_2.5_ occurred when one or more days during this time period was higher than the average for 1–7 November 2018 (pre-wildfire period).

### 2.4. Outcomes

Mental health symptoms were self-reported perceived changes in symptoms since the wildfire. Outcomes were measured as the total count of nine symptoms: agitated behavior; anxiety or stress; depressed mood; difficulty concentrating; loss of appetite; tobacco or vaping use; alcohol or drug use; trouble sleeping or nightmares; and withdrawal from daily activities. The survey, administered only once, asked about occurrence of these symptoms at three weeks, one month, three months, and six months after Camp Fire (Supplementary Table S1).

### 2.5. Exposures and Covariates

We defined our exposures as wildfire related factors: home damage during the wildfire, loss of a family member or friend, loss of job and loss of a pet due to wildfire. The home damage variable had four categories (not damaged, minor damage, still standing but significantly damaged, and completely destroyed). Because of small numbers of households with significant damage, we combined this category with those that were completely destroyed. Fire-related losses were coded as a binary variable (yes/no).

Covariates included age (0–18, 19–34, 35–64, ≥65 years), sex (male, female), race/ethnicity (Hispanic, Non-Hispanic White, Multiracial, Other), prior asthma, prior physician diagnosis of a mental health condition, prior physical disability, home damage during the wildfire, and living alone. For the socioeconomic status (SES) variable we calculated an area-level composite index utilizing U.S. Census data for the time period: percent unemployed, percent Hispanic population, percent of residents without secondary education, and percent of owner-occupied housing. For each of these four indicators, the block group for each household was categorized into county-level quintiles and assigned ranks from zero to four, with zero being the quintile with the greatest ‘disadvantage’. Ranks were summed with equal weighting across four indicators to create a composite score ranging from zero to 16, with lower scores indicating greater vulnerability. We operationalized SES in the analysis by creating cohort-based quartiles.

### 2.6. Statistical Analysis

To identify risk factors for mental health symptom counts at week three, we employed a hurdle negative binomial model within a generalized linear mixed-effects modeling framework. This approach is appropriate for overdispersed count data with excess zeros. The model comprises a logistic hurdle component that estimates the probability of zero versus nonzero symptom counts and a truncated negative binomial component that models positive counts only [23,24,25,26].

To examine changes in survivors’ mental health symptoms over a six-month period following exposure to the Camp Fire, we compared Poisson, negative binomial, zero-inflated Poisson, zero-inflated negative binomial, hurdle Poisson, and hurdle negative binomial models. The hurdle negative binomial model was selected because it demonstrated the lowest dispersion and the most favorable Akaike Information Criterion (AIC) and Bayesian Information Criterion (BIC) statistics. Thus, two kinds of output are produced: For the positive symptom count (conditional on having one or more symptoms), rate ratios are obtained to represent the ratio of expected symptom counts for participants with one or more symptoms, comparing a category of interest to the referent group; for the binary variable (one or greater vs. zero), the output is an Odds Ratio, interpreted as a logistic regression, a comparison of the odds for the 1+ vs. zero groups. To account for the clustering effect of households, a random intercept for household ID was included. We included an interaction term between the covariates and time variable to evaluate how symptom trajectories varied over the study period. Covariates included in the analytic model were selected based on previously published literature, a directed acyclic graph (DAG) constructed using prior knowledge, and a stepwise regression approach with decisions based on change-in-estimate.

To address missing data for covariates, we performed multiple imputations by chained equations (MICE) with nine imputations and five iterations per imputation [27]. Missing data were imputed for the following covariates: (a) age (9.2% missing); (b) race (9.0% missing); (c) sex (3.5% missing); (d) socioeconomic status (SES) (2.7% missing); (e) prior asthma (1.9% missing); (f) prior mental health condition (1.9% missing); (g) prior cancer (1.9% missing), (h) prior physical-disability (1.9% missing), (i) wildfire-related home damage (2.5% missing), (g) evacuation status (0.8% missing) and (k) mental health symptoms outcome (1.3% missing).

## 3. Results

There were 4346 survey participants included in the study. Table 1 provides summary statistics on sociodemographic factors, wildfire-related characteristics, prior health conditions, and evacuation status. The cohort predominantly comprised individuals aged 35 to 64 years (44.5%), with a higher representation of females (55.6%) and 82.6% identifying as non-Hispanic White. Pre-existing health conditions were as follows: 14.5% of survivors with prior asthma, 10.2% with prior psychiatric/mental health conditions, and 6.2% with physical disabilities. Almost half of the study population evacuated (51%), and approximately one-quarter of homes were either completely destroyed or significantly damaged (Table 1).

**Figure 2:**
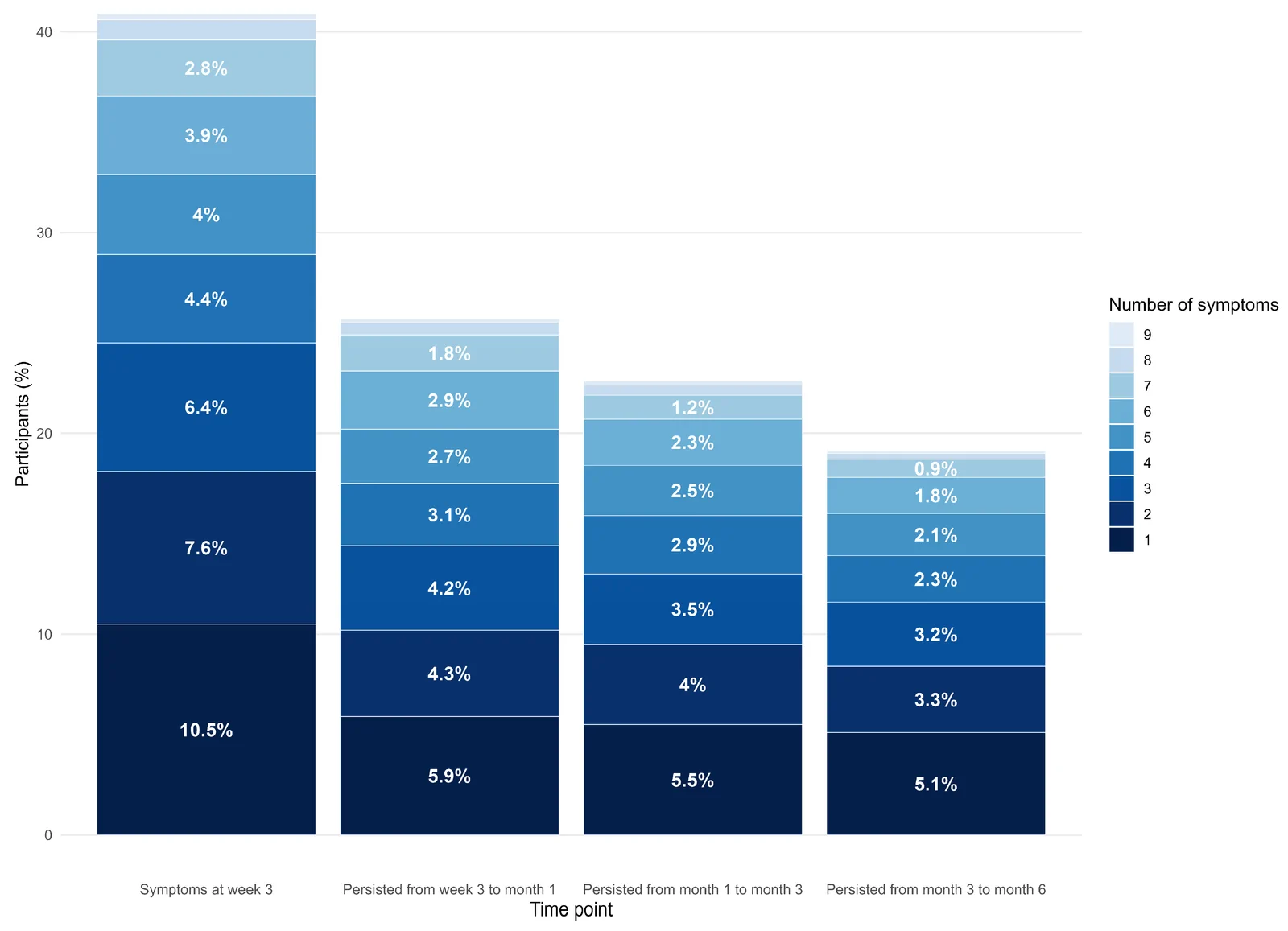
Persistence of mental health symptoms over six months. The denominator for all percentages is the study cohort (N = 4346). Persistence was defined as the same symptom being present at the specified and preceding points.

Approximately 41% of survivors reported at least one mental health symptom at three weeks. The persistence of the same symptom over consecutive time points declined between week three and month six. At month six, persistence of any individual symptom was reduced by nearly half (10.5% vs. 5.1%), with comparable declines observed for multiple symptoms (Figure 2, Supplementary Table S2).

In the immediate aftermath of the fire, among survivors with one or more mental health symptoms, after multivariate adjustment, survivors under age 18 experienced a 21% lower (RR = 0.79, 95% CI: 0.70, 0.91) rate of mental health symptoms, compared to those age 18–34. Male sex was associated with a 16% lower (RR = 0.84, 95% CI: 0.79, 0.90) rate of mental health symptoms, as compared with females. The rates of mental health symptoms were higher for survivors with prior physical disability (RR = 1.13, 95% CI: 1.01, 1.27), survivors with prior mental health condition (RR = 1.22, 95% CI: 1.11, 1.34), survivors whose home was either destroyed or significantly damaged (RR = 1.61, 95% CI: 1.43, 1.81) and those with minor damage due to the Camp Fire (RR = 1.25, 95% CI: 1.06, 1.48), loss of a family member or friend (RR, 1.14; 95% CI, 1.02–1.28), and job loss (RR, 1.17; 95% CI, 1.07–1.27) (Table 2).

Results from the adjusted binary component of the hurdle negative binomial model which estimates the odds of having one or more mental health symptoms versus none, showed that survivors aged 36–64 years and 65 years or older had higher odds of having one or more mental health symptoms vs. none compared with those aged 18–34 years (OR, 1.32; 95% CI, 1.12–1.56 and OR, 1.32; 95% CI, 1.06–1.65, respectively). Male sex was associated with 0.61 (OR, 0.61; and 95% CI: 0.54–0.67) times the odds of having one or more vs. no symptoms compared with females.

Prior mental health condition (OR = 1.49; 95% CI, 1.20–1.84), living alone (OR = 1.35, 95% CI: 1.03–1.78), and evacuation (OR, 1.35; 95% CI, 1.10–1.68) were each associated with a higher odds of having one or more vs. no symptoms. Survivors whose homes were completely destroyed or severely damaged (OR = 1.63; 95% CI: 1.26–2.11) compared with those whose homes were not damaged had a higher odds of having one or more vs. no symptoms (Table 2).

**Figure 3:**
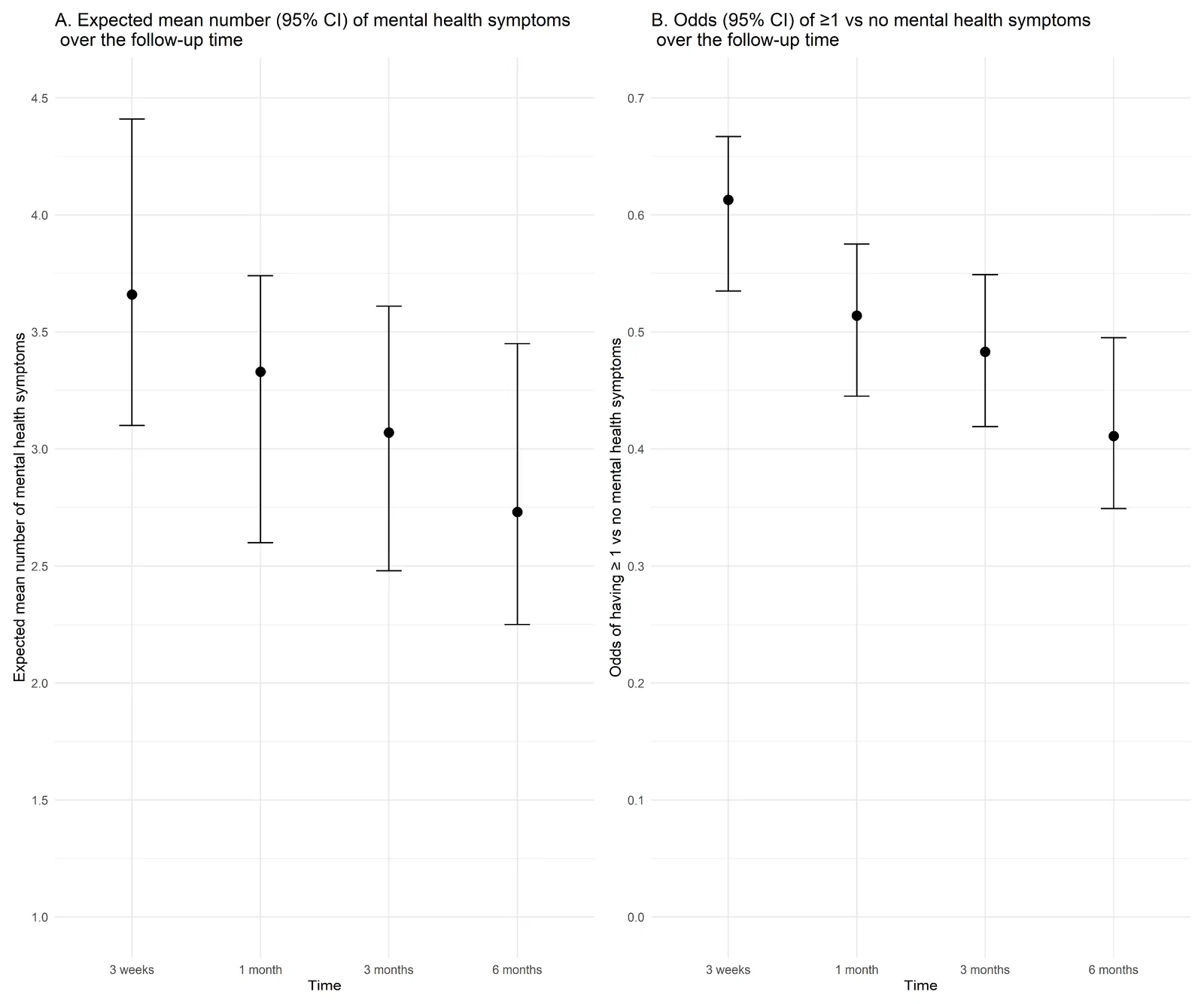
Mental health symptoms over the follow-up time from adjusted mixed effects hurdle negative binomial model. (**A**) Results from the count components of mixed effects hurdle negative binomial model, adjusted for age, sex, race, SES, prior psychiatric or mental health conditions, physical disability, home damage, loss of family or friend, loss of pet, loss of job, and time. (**B**) Results from the binary component of mixed effects hurdle negative binomial model, adjusted for age, sex, race, SES, prior psychiatric or mental health conditions, physical disability, home damage, loss of family or friend, loss of pet, loss of job, and time.

Among survivors with symptoms, the average number of mental health symptoms decreased over the follow-up period: three weeks, 3.7 (95% CI, 3.3–4.1); 1 month, 3.3 (95% CI, 3.0–3.7); three months, 3.1 (95% CI, 2.8–3.4); and 6 months, 2.7 (95% CI, 2.46–3.1) (Figure 3A). Similarly, the odds of having one or more vs. no mental health symptoms decreased over time (Figure 3B).

**Figure 4:**
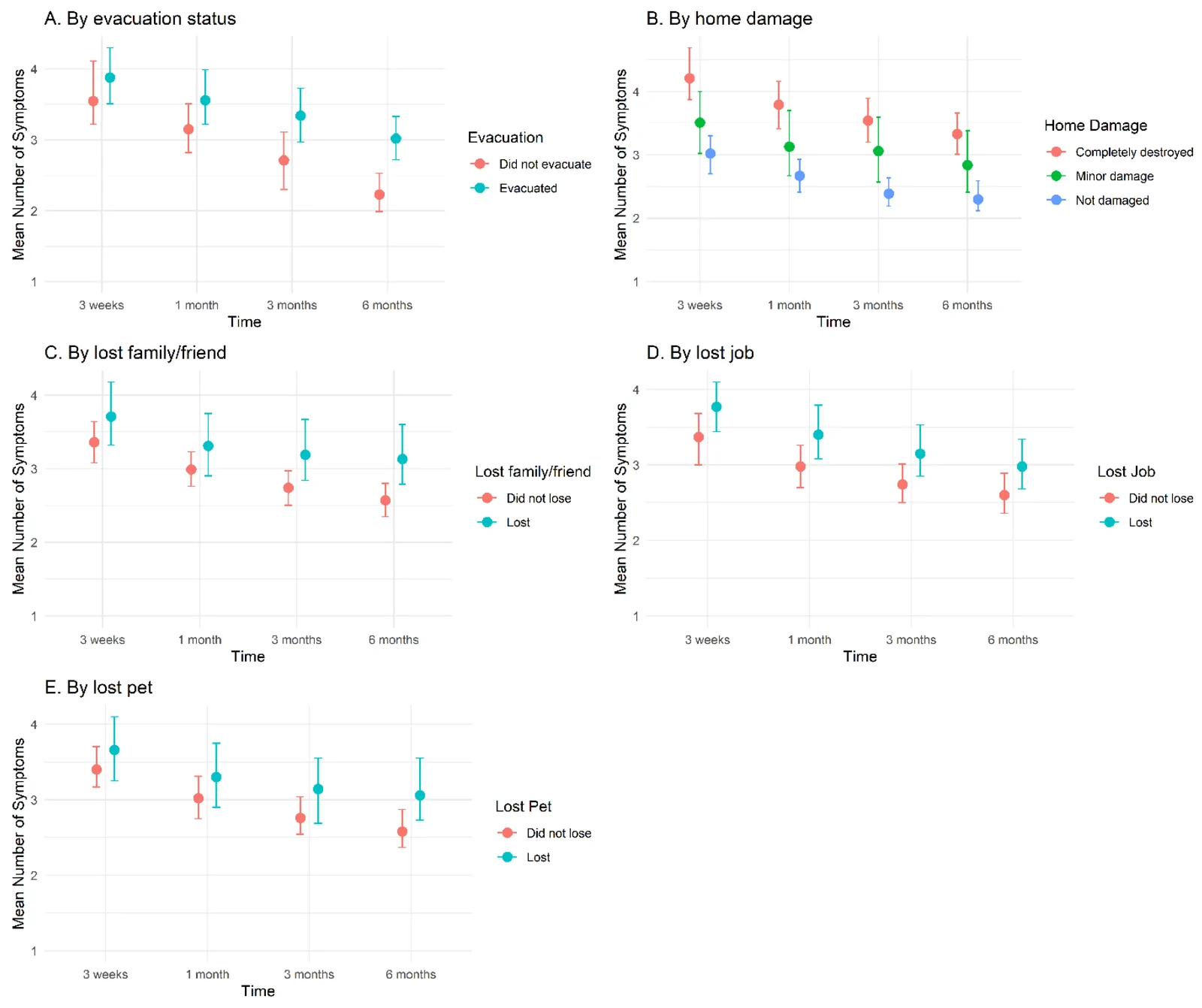
Mean number of mental health symptoms (95% CI) over the follow-up time, by wildfire-related experiences and losses. Results are from the count component of mixed effects hurdle negative binomial models. Each model adjusts for age, sex, race, socioeconomic status (SES), prior psychiatric or mental health conditions, physical disability, evacuation status, and home damage, as well as the interaction between each wildfire-related exposure or loss variable and time, with separate models for each interaction.

A downward trend over time was observed for wildfire-related characteristics and losses. In stratified models, evacuees had a slightly higher rate of mental health symptoms at three weeks compared with non-evacuees. This difference diminished over time, with non-evacuees clearly exhibiting a steeper decline in symptoms (Figure 4A). Those with completely destroyed and not damaged homes showed a sharper decline compared with those who had minor damage (Figure 4B).

Loss of a family member or friend, job loss, and pet loss showed similar declines across all groups, with most group differences remaining relatively stable over time (Figure 4C–E).

**Figure 5:**
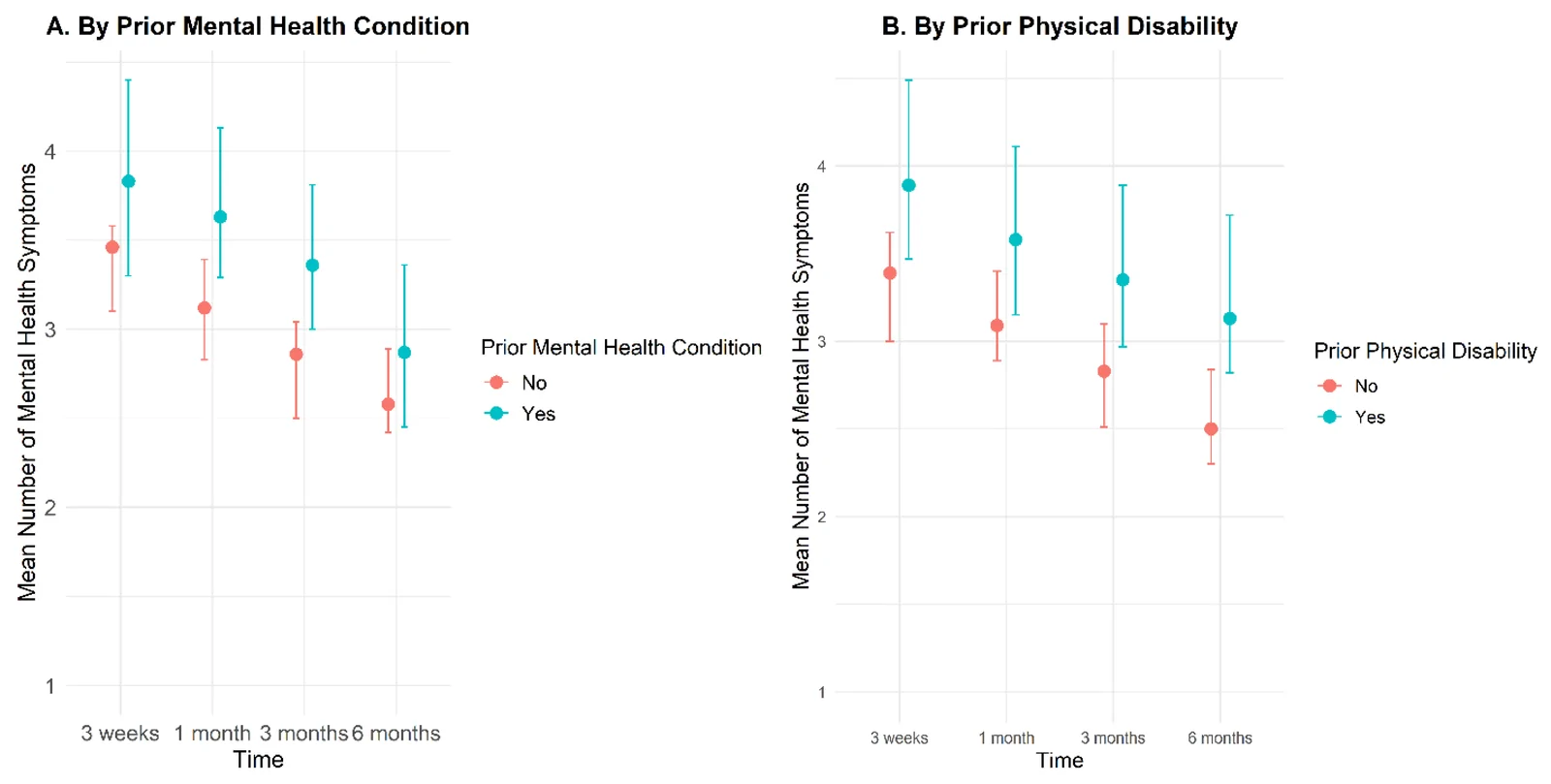
Mean number of mental health symptoms (95% CI) over the follow-up time, by prior comorbidities. Results are from the count component of mixed effects hurdle negative binomial models. Each model adjusts for age, sex, race, SES, evacuation status, home damage, and the interaction between each comorbidity and time, with separate models for each interaction.

At three weeks following the Camp Fire, survivors with a history of mental health conditions or physical disabilities reported a higher number of mental health symptoms compared to those without prior comorbidities. Over time, the number of symptoms declined across all groups. Notably, individuals with prior mental health conditions demonstrated a sharper reduction in symptoms between three and six months post-disaster compared to those without prior mental health conditions. Those with prior physical disability had higher rate of mental health symptoms at all four time periods, and decline over time was similar to those without physical disability (Figure 5A,B).

## 4. Discussion

This study investigated both the acute mental health impacts of wildfires and the trajectories of symptoms over six months of follow-up. In contrast with other publications on this topic, the current study collected detailed data on a wide array of specific wildfire-related experiences and on pre-existing conditions. This information allowed us to quantitate the differential impacts of these personal wildfire-induced events (evacuations, losses, etc.) and of prior health conditions or living situation on the individual’s burden of mental health disturbances after the fires. This approach highlights the main drivers of mental health changes, and furthermore captures the changes over the first six months post-wildfire.

In the acute phase, about 41% of survivors experienced at least one symptom, and approximately half of the same symptoms persisted at six months (Figure 2). The findings of acute impacts indicate that sex, complete loss of home or significant home damage (vs. no damage), loss of a family member or friend, job loss, pet loss, self-reported prior physician diagnosis of a mental health condition, and prior physical disability were significant predictors of mental health symptoms. These findings are consistent with the broader disaster mental health literature, which suggests that both losses due to disaster exposure and pre-existing individual vulnerabilities influence psychological outcomes. Property losses, such as severe home damage, may contribute to psychological distress by disrupting housing stability, daily routines, and an individual’s sense of safety and control [28]. Similarly, bereavement following a disaster represents a profound interpersonal loss that may compound the psychological impact disrupting emotional support networks, increasing feelings of helplessness, and complicating recovery [29,30]. Individuals with pre-existing mental health conditions or physical disabilities may have fewer psychological and physical resources available to cope with disaster-related stressors and may experience greater challenges accessing healthcare, social services, and community resources during recovery [31,32].

The finding that individuals younger than 18 years of age reported fewer mental health symptoms than young adults was unexpected, as previous studies have generally found that children and adolescents are particularly vulnerable to adverse psychological outcomes following wildfire disasters, including elevated symptoms of post-traumatic stress, anxiety, and depression [9,33]. One explanation for why individuals under 18 years of age had fewer reported mental health symptoms is that one adult respondent reported information on behalf of all household members, and the respondent may have been projecting their own mental health status or may not be fully aware of the mental health status of children and adolescents in the household (Table 2).

Findings from the six-month follow-up suggest a decreasing trajectory in mental health symptoms among both evacuees and non-evacuees, with much steeper decline in the non-evacuated subgroup from three to six months. Symptom trajectories over these periods, among those with prior comorbidities and wildfire-related losses (e.g., loss of a family member or friend, pet, or job) also showed declines compared with the acute phase, however at month sex, 19.2% had at least one persistent mental health symptom (Supplementary Table S2).

This study has several limitations. First, data collection relied on self-reported information, with 85% of responses collected between 6–8 months. Consequently, recall bias may have occurred, as participants may have had difficulty accurately remembering past experiences or symptoms, particularly for specific, narrowly defined retrospective time periods. Moreover, traumatic memories are reconstructive rather than static and recall of earlier psychological symptoms may be influenced by current emotional state, collective memory, and the broader social discourse surrounding the disaster [34,35,36]. These biases are in unknown direction and the net bias across the population may not be possible to project, however our sample (restricted to Butte County) comparison with the Butte County population was very similar in terms of prior mental health/psychiatric history (13% vs. 14.6% Supplementary Table S3). Second, due to recruitment relying heavily on Facebook wildfire groups, there is potential for selection bias related to language, socioeconomic status and age. However, the comparison of our study sample from Butte County with the Butte County population (the location of Camp Fire) showed very similar age and prior comorbidity distributions (Supplementary Table S3).

Although a Spanish-language version of the survey was available, the use of Facebook in Spanish was still emerging during that period, which may help explain the lower participation of Spanish speakers. Given that 56% of our study sample resided in Butte County, we compared ethnic and racial distribution of our sample with Butte County population data. We acknowledge the underrepresentation of White Hispanics and races other than White or multiracial. The Non-Hispanic Whites were overrepresented in this sample, whereas multiracial group was appropriately represented based on Butte County data. (Supplementary Table S3). In addition, individuals with major chronic health conditions or those who experienced severe mental health trauma may have been underrepresented in the study population. Individuals most severely affected or traumatized by the wildfire may have been unable to complete the survey, resulting in underestimation of the true impact. Conversely, convenience sampling may have resulted in oversampling individuals who experienced greater distress or had a stronger interest in wildfire-related health issues, therefore, given potentially opposite trends that might be occurring simultaneously, the directionality of any bias and its magnitude remains unknown.. Third, there is potential for reporting bias, as one household member completed the survey on behalf of all household members, particularly children and adolescents. This approach may have reduced the accuracy of individual-level data, as adults may not be fully aware of the mental health status of children and adolescents and may project their own experiences or perceptions onto other household members.

Fourth, the outcomes were based on self-reported symptoms rather than objective diagnoses or validated questionnaires. This approach limits our ability to directly compare our findings with studies that use standardized instruments and may result in higher rates of mental health distress in our population. However, we adopted this symptom-based approach to capture a broader spectrum of mental health impairments, including subclinical symptoms that may not meet formal diagnostic criteria but can nevertheless adversely affect survivors’ daily functioning and quality of life.

This study has several strengths. First, our approach captures impairments that do not meet formal diagnostic criteria, symptoms in individuals who do not seek care, and experiences of those for whom medical services were unavailable due to wildfire-related destruction of major hospitals. Second, this study provides important insights into the burden of wildfires on survivors’ mental health, as reliance on clinical diagnoses alone may underestimate the psychological impact of disaster exposure. In population-based settings, many individuals experience increases in distress, sleep disruption, cognitive difficulties, and behavioral changes that do not meet diagnostic thresholds or do not lead to healthcare seeking. These subclinical symptoms are nonetheless associated with functional impairment, reduced quality of life, and have been shown to predict the subsequent development and persistence of diagnosable mental health disorders [17,18,19,20,21].

Third, the current study provides valuable insights into the predictors and vulnerability factors of mental health symptoms in the aftermath of wildfires by examining a range of baseline comorbidities, sociodemographic factors, and wildfire-related variables. Fourth, this project significantly advances prior understanding by investigating the trajectories of mental health symptoms across multiple time points—week three, one month, three months, and six months post-wildfire—contrasting with previous research focused on single follow-up time points.

Fourth, to our knowledge, this study is one of the largest questionnaire-based investigations of mental health symptoms to date that examined a wide range of sociodemographic variables, prior comorbidities, wildfire-related experiences and losses, with a sample of 4356 participants [6,12,16,37]. Prior questionnaire-based studies were typically smaller and were limited to evacuees, without comparing the experiences of evacuees with those who did not evacuate. For instance, a study of the 2016 Fort McMurray wildfires reported elevated levels of PTSD, depression, and insomnia among evacuees three months post-disaster [16], based on a sample of 399 evacuees. Another investigation into the aftermath of the McMurray wildfires documented a 12.6% prevalence of probable PTSD six months post-event, drawing from a sample of 488 individuals. Additionally, another study of Fort McMurray wildfire survivors involving 290 respondents found high prevalence rates of mental health and addiction conditions among patients attending out-of-hours clinics at 18 months following the disaster [12]. More recently, a study of 1174 survivors of the 2023 Maui wildfires reported that nearly half of respondents (49.4%) screened positive for depressive symptoms, and over one quarter (27.2%) reported clinically relevant anxiety symptoms 6–14 months post-wildfire [38]. A further strength of this study is that we examined a wide range of comorbidities and wildfire-related experiences that are rarely included in previous research, including evacuation, degree of home damage, and losses of family members, friends, pets, or jobs.

It is notable that some of the greatest health needs among survivors of the 2017 California wildfires include both acute mental health needs in the immediate aftermath of the wildfires (i.e., during the week following the wildfire) and persistent mental health needs four to nine months post-wildfire [7]. Our findings add further evidence regarding the importance of resources to address these highly prevalent acute mental health needs. In the future, we will report on a longer-term follow-up of those who experienced wildfire-related mental health symptoms.

## 5. Conclusions

This study provides valuable insights into the acute mental health impacts of the 2018 Camp Fire using a symptom-based approach and identifies vulnerable subgroups, including individuals who experienced greater home damage or loss of family, friends, pets, or jobs. It suggests the potential value of mental health monitoring and early support to prevent long-term mental health symptoms, particularly for those in vulnerable groups. By capturing mental health burden through symptom-based measures rather than relying solely on binary diagnostic outcomes, this study suggests the need for early public health services and interventions that may mitigate progression to more severe mental health conditions.

## Figures and Tables

**Table 1:** Socio-demographic, wildfire-related, and prior health characteristics of the cohort at baseline.

Characteristic	N = 4346 ^1^
Age (years)	
18–34	835 (19.2%)
<18	798 (18.4%)
35–64	1936 (44.5%)
65+	777 (17.9%)
Sex	
Female	2418 (55.6%)
Male	1928 (44.4%)
Race	
Non-Hispanic White	3591 (82.6%)
Multiracial	280 (6.4%)
White Hispanic	276 (6.4%)
Other ^2^	199 (4.6%)
Socioeconomic status quartile	
Q1 (Lowest SES)	1087 (25.0%)
Q2	1087 (25.0%)
Q3	1086 (25.0%)
Q4 (Highest SES)	1086 (25.0%)
Live alone (Yes)	269 (6.2%)
Prior asthma (Yes)	628 (14.5%)
History of mental health condition(s) (Yes)	442 (10.2%)
Prior physical disability (Yes)	268 (6.2%)
Evacuation status (Yes)	2221 (51.1%)
Wildfire-related home damage	
Not damaged	2846 (65.5%)
Completely destroyed or significantly damaged	1159 (26.7%)
Minor damage	341 (7.8%)
Loss of family member or friend (Yes)	468 (10.8%)
Lost job (Yes)	1253 (28.8%)
Lost pet (Yes)	580 (13.3%)

^1^ n (%). ^2^ “Other” race and ethnicity included American Indian or Alaska Native, Asian and Black.

**Table 2:** Adjusted associations between participant characteristics and mental health symptoms at week 3 ^1^ (N = 4346).

Characteristic	Count Component		Logistic Component (Odds of Having ≥1 vs. No Symptom	
	RR (95% CI) ^2^	*p*-Value	OR (95% CI) ^3^	*p*-Value
*Socio-demographics*				
Age				
<18	0.79 (0.70–0.91)	<0.001	0.79 (0.65–0.97)	0.024
18–34 (ref.)				
35–64	1.00 (0.92–1.10)	0.948	1.32 (1.12–1.56)	0.001
65+	0.92 (0.82–1.03)	0.162	1.32 (1.06–1.65)	0.015
Sex				
Female (ref.)				
Male	0.84 (0.79–0.90)	<0.001	0.61 (0.54–0.67)	<0.001
Race				
Non-Hispanic White (ref.)				
White Hispanic	0.86 (0.72–1.02)	0.074	1.29 (0.99–1.68)	0.057
Multiracial	1.08 (0.94–1.24)	0.267	0.80 (0.60–1.07)	0.140
Other ^4^	1.06 (0.87–1.28)	0.572	0.84 (0.62–1.14)	0.300
SES				
Q1 (Lowest SES) (ref.)				
Q2	0.97 (0.87–1.09)	0.638	0.75 (0.59–0.95)	0.016
Q3	1.00 (0.90–1.13)	0.938	0.95 (0.76–1.20)	0.700
Q4 (Highest SES)	0.90 (0.80–1.01)	0.071	0.77 (0.62–0.97)	0.025
Living alone (Yes)	1.05 (0.92–1.19)	0.486	1.35 (1.03–1.78)	0.031
*Baseline Comorbidities*				
History of mental health condition (s) (Yes)	1.22 (1.11–1.34)	<0.001	1.49 (1.20–1.84)	<0.001
Prior physical disability (Yes)	1.13 (1.01–1.27)	0.033	1.27 (0.98–1.65)	0.071
*Wildfire-related characteristics and loses*				
Evacuated (Yes)	1.10 (0.98–1.23)	0.121	1.35 (1.10–1.68)	0.003
Home damage				
Not damaged (ref.)				
Completely destroyed or significantly damaged	1.61 (1.43–1.81)	<0.001	1.63 (1.26–2.11)	<0.001
Minor damage	1.25 (1.06–1.48)	0.007	1.28 (0.91–1.79)	0.200
Lost a family member or friend (Yes)	1.14 (1.02–1.28)	0.023	1.36 (1.03–1.78)	0.028
Lost pet (Yes)	1.12 (1.01–1.26)	0.038	1.11 (0.85–1.46)	0.400
Lost job (Yes)	1.17 (1.07–1.27)	<0.001	1.20 (1.01–1.43)	0.033

^1^ Results are estimated using a mixed-effects hurdle negative binomial model, adjusted for the clustering effect of households and the covariates listed above. ^2^ RR = rate ratio, CI = confidence interval from the count component of the mixed-effects hurdle negative binomial model. ^3^ OR = odds ratio, CI = confidence interval from the binary logistic component of the mixed-effects Hurdle Negative binomial model, modelling the probability of having ≥1 vs. no health symptoms. ^4^ “Other” race and ethnicity included American Indian or Alaska Native, Asian, and Black.

## Data Availability

The data will be made available in accordance with federal guidelines and requirements for Data Sharing.

## Supplementary Materials

The following supporting information can be downloaded at https://www.mdpi.com/article/10.3390/ijerph23081005/s1, Table S1: Questions from the baseline survey to measure changes in mental health symptoms; Table S2: Persistence of Mental Health Symptoms Over 6 Months of Follow-up; Table S3: Sociodemographic, Wildfire-related Characteristics and Pre-Wildfire Comorbidities Among Baseline Survey Respondents in Butte County Compared with County-Level Estimates [39].
