# Supplementary material for: Acute Mental Health Impacts and Mid-Term Symptom Trajectories Following Wildfire Exposure
Source: Int J Environ Res Public Health. 2026 Jul 31;23(8):1005. doi: 10.3390/ijerph23081005 (PMC13512769; doi:10.3390/ijerph23081005)
Supplement: Supplementary file 1 [file ijerph-23-01005-s001.zip › ijerph-4364902-supplementary.pdf]

**The following table was used in our baseline survey to collect data for each mental health symptom and their timing**

**Supplementary Table S1. Questions from the baseline survey to measure changes in mental health symptoms.**

| Compared to before the fire, did you have a greater level or frequency in any of the following? | | | | |
|---|---|---|---|---|
| | First 3 weeks after the fire | 1 month after the fire | 3 months after the fire | 6 months after the fire |
| Agitated behavior | | | | |
| Anxiety or stress | | | | |
| Depressed mood | | | | |
| Difficulty concentrating | | | | |
| Loss of appetite | | | | |
| Trouble sleeping or nightmares | | | | |
| Alcohol or drug use | | | | |
| Smoking (tobacco or vaping) | | | | |
| Withdrawn from daily activities | | | | |

**Supplementary Table S2. Persistence of Mental Health Symptoms Over 6 Months of Follow-up**

| | | | | |
|---|---|---|---|---|
| **Number of symptoms** | ***Symptoms at week 3 N (%)[1]*** | ***Persisted[2] from week 3 to month 1 N (%)*** | ***Persisted[2] from month 1 to month 3 N (%)*** | ***Persisted[2] from month 3 to month 6 N (%)*** |
| 1 symptom | 458 (10.5%) | 255 (5.9%) | 239 (5.5%) | 222 (5.1%) |
| 2 symptoms | 331 (7.6%) | 188 (4.3%) | 176 (4.0%) | 143 (3.3%) |
| 3 symptoms | 279 (6.4%) | 182 (4.2%) | 150 (3.5%) | 139 (3.2%) |
| 4 symptoms | 193 (4.4%) | 136 (3.1%) | 126 (2.9%) | 98 (2.3%) |
| 5 symptoms | 176 (4.0%) | 119 (2.7%) | 110 (2.5%) | 91 (2.1%) |
| 6 symptoms | 169 (3.9%) | 125 (2.9%) | 99 (2.3%) | 78 (1.8%) |
| 7 symptoms | 121 (2.8%) | 79 (1.8%) | 54 (1.2%) | 41 (0.9%) |
| 8 symptoms | 42 (1.0%) | 24 (0.6%) | 23 (0.5%) | 15 (0.3%) |
| 9 symptoms | 12 (0.3%) | 9 (0.2%) | 7 (0.2%) | 6 (0.1%) |
| At least 1 symptom | 1,781 (41.0%) | 1,117 (25.7%) | 984 (22.6%) | 833 (19.2%) |

[1]% of the study cohort (N=4,346)

[2]Persistence was defined as the same symptom being present at the specified and preceding time points

**Supplementary Table S3: Sociodemographic, Wildfire-related Characteristics and Pre-Wildfire Comorbidities Among Baseline Survey Respondents in Butte County Compared with County-Level Estimates**

| | **Baseline survey, N = 2,267[1]** | **Butte County, N = 220.000[2]** |
|---|---|---|
| **Age (years)** | | |
| <18 | **20.0%** | **20.4 %** |
| 18-64 | **63.9%** | **60.8%** |
| 65+ | **18.1%** | **18.8%** |
| **Sex** | | |
| Female | **55.6%** | **50.4%** |
| Male | **44.4%** | **49.6%** |
| **Race and Ethnicity** | | |
| Non-Hispanic White | **83.8%** | **66.4%** |
| Multiracial | **6.5%** | **5.2%** |
| White Hispanic | **6.3%** | **20.6%** |
| Other | **4.4%** | **7.8%** |
| **Prior asthma (Yes)** | **14.7%** | **15.0%** |
| **Prior diabetes (Yes)** | **25.2%** | **23.5%** |
| **Median household size** | **3.00** | **3.00** |
| **Prior metal health/ psychiatric disorder (Yes)** | **13.0%** | **14.62%[c]** |

**[1]Baseline survey restricted to Butte County respondents only.**

**[2] Butte County data were derived from either the 2010 U.S. Census or the 2018 Behavioral Risk Factor Surveillance System (BRFSS). Data on age, sex, race, and median household size came from the U.S. Census, while data on smoking status, prior asthma, and diabetes were obtained from the BRFSS.**

**[3] Based on whether the individual saw a primary care provider or specialist for mental health (Eberhart et al, 2019).**[39]

**3)** Eberhart, N.K.; Burnam, M.A.; Seelam, R.; Bogdan, O.; Breslau, J. Monitoring Californians' Mental Health: Population Surveillance Reveals Gender, Racial/Ethnic, Age, and Regional Disparities. *Rand Health Q.* **2019**, *8*, 5.
